# Non-Conventional Methods for Gelation of Alginate

**DOI:** 10.3390/gels4010014

**Published:** 2018-02-01

**Authors:** Pavel Gurikov, Irina Smirnova

**Affiliations:** Institute of Thermal Separation Processes, Hamburg University of Technology, Eißendorfer Straße 38, 21073 Hamburg, Germany; irina.smirnova@tuhh.de

**Keywords:** alginate, cryogelation, cryogel, aerogel, macroporosity, supercritical drying, freeze drying

## Abstract

This review presents and critically evaluates recent advances in non-conventional gelation method of native alginate. A special focus is given to the following three methods: cryotropic gelation, non-solvent induced phase separation and carbon dioxide induced gelation. A few other gelation approaches are also briefly reviewed. Results are discussed in the context of subsequent freeze and supercritical drying. The methods are selected so as to provide the readers with a range of novel tools and tactics of pore engineering for alginate and other anionic polysaccharides.

## 1. Introduction

**Figure 1:**
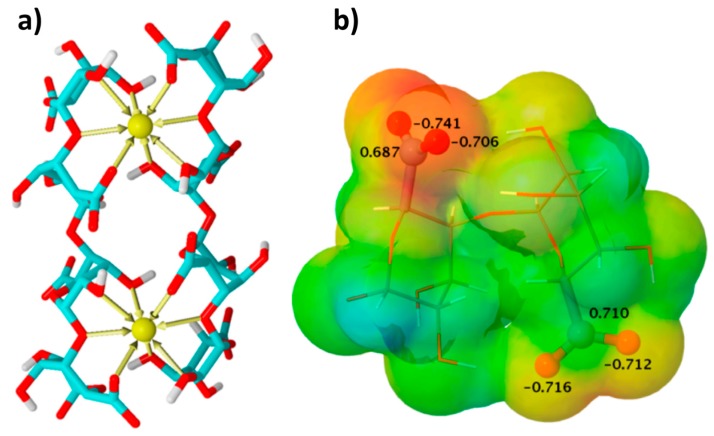
The classical egg-box model (**a**); the charge distribution of the α-l-guluronate anion, G residual (**b**). Adapted with permission from Plazinski and Drach [3]. Copyright (2013), American Chemical Society.

Alginates is a family of linear copolymers with homopolymeric blocks of (1,4)-linked β-d-mannuronate (M) and its C-5 epimer α-l-guluronate (G) residues. The M and G residuals are linked together in different sequences. It is well known that monovalent salts of alginates undergo gelation in the presence of divalent and trivalent cations. The G residues are believed to be predominantly responsible for binding the M^n+^ cations through the so-called egg-box model (Figure 1a). According to this model, two facing helical stretches of G sequences bind the divalent ion in a chelate type of binding [1]. Since its development in 1970s, many refinements of the model have been suggested in the literature (see, for example, [2,3] and references therein).

There are two conventional ways to gel alginates [4]: by lowering pH below the pKa values (3.4 and 3.6 for M and G units) and by introducing cations, i.e., through ionotropic gelation. The ionotropic gelation can be realized as the diffusion setting or the internal setting. In the simplest version of the diffusion setting, a solution of alginate salt (most often Na-alginate) is extruded dropwise into a gelation bath with a soluble calcium salt, e.g., CaCl_2_. Gelation occurs rapidly preserving spherical shape of the droplets. In the internal setting method, the gelation is mediated by a change in pH, which, in its turn, releases crosslinking cations from insolubility of a chelated form. Calcium carbonate with slowly hydrolyzing d-glucono-δ-lactone (GDL) is one of the most studied systems.

The focus of the review is on non-conventional gelation methods for native alginate. They are merely presented by a few examples in the literature, but, in our eyes, offer flexible and auspicious approaches for the engineering of porous alginate-based materials. Because wet gels are often the only starting materials, we also discuss the non-conventional gelation methods with respect to subsequent drying. As evaporative drying is an inappropriate method for the preservation of delicate gel structures, especially for biopolymer gels, only supercritical drying and freeze drying are discussed. We put aside gelation routes for alginate derivatives as well as those studies where a second component is responsible for the formation of a hybrid gel. Wherever available, the alginate characteristics such as molecular weight and the G/M composition are given (in the form identical to the original publication).

## 2. Cryogelation of Alginate

Cryotropic gelation (or cryogelation) consists of three steps: non-deep freezing of a solution with monomeric or polymeric precursors, and its storage in the frozen state followed by thawing. Gels formed under such conditions are known as cryogels [5]. In this review, the term “cryogel” is used for gels formed as a result of non-deep freezing of a polymer solutions or a colloidal sol followed by thawing. Cryogels possess a large fraction of interconnected macropores and may even have so-called gigapores (~10–100 µm). Such pores are formed by growing crystals of the solvent. In the current literature, the term “cryogel” often has a different meaning, designating a solid material formed by the removal of all swelling agents from a gel by freeze drying. In this case, the method of how such gel is obtained is immaterial. 

### 2.1. Cation-Free Cryogelation

Systematic studies on cryogelation of polymers such as polyvinyl alcohol (PVA) and crosslinked polyacrylamide began in the 1970s. Nowadays, the mechanism and influencing parameters for such systems are well elucidated [5,6]. Basically, the mechanism relies on the existence of the unfrozen liquid microphase in non-deep frozen polymer solutions. This phase acts as a microreactor where polymer chains (and if needed crosslinkers) are concentrated and can form physical or chemical gels upon thawing [6]. 

There is considerably less known about cryogelation of biopolymers, in particular anionic polysaccharides. In the case of xanthan gum, the mechanism is believed to be analogous to cryogelation of PVA, where xanthan chains are forced to concentrate, align and associate in liquid domains between growing ice crystals. The forced associations survive upon thawing, yielding the cryogel network [7]. For other reported anionic polysaccharides (sodium hyaluronate, carboxymethyl curdlan and carboxymethyl cellulose), protonation by lowering the pH is an essential prerequisite for the association of chains and thus successful cryogelation [8]. 

Thus, the cryogelation of some anionic polysaccharides have been demonstrated [8]; until recently, alginate was an exception. To the best of our knowledge, Florián-Algarín and Acevedo [9] were the first who reported the existence of cryogelation for sodium alginate solutions. The authors applied the constant-stress temperature-ramp viscosity method, the oscillatory dynamic test (viscoelastic moduli were measured while cooling) and the thixotropy test (steady-state viscosity as a function of the shear rate below and above *T*_gel_). The results clearly indicated that thermally reversible gelation of sodium alginate occurred at 15–16 °C (1.5 wt %, *M*_w_ = 291 kDa, M content 39 ± 4%); however, the resulting gels were very weak. It is important for the discussion below that the measurements were performed at pH 5.2–5.4.

The first self-supporting alginate cryogels were reported by Zhao et al. [10]. It was demonstrated that storage of sodium alginate solution (0.5–1.0 wt %, *M*_w_ = 80–120 kDa, M/G ratio 1.56) at −25 °C for 24 h followed by thawing at 4 °C for another 24 h resulted in weak gels. The key parameter of the process is the pH: cryogels could be obtained at any pH value between 2.0 and 4.0 after one or few freezing-thawing cycles, whereas acidified alginate solutions resulted in soft “flowable” gels only in the pH range of 2.5–3.0. No gel formed even after five freezing-thawing cycles at pH 4.5–5.0 [10].

These findings can be interpreted as a proper extent of polymer association (a balance between repulsive and attractive interactions) at a lowered pH (≤4.0) being required for the formation of junction zones through van der Waals forces and hydrogen bonds between partially protonated (uncharged) alginate chains in unfrozen liquid microphase. This interpretation explains why cryogelation was observed only below a certain pH that is close to the pKa values of alginate. These results may also explain why the gel formation was detected by Florián-Algarín and Acevedo only rheologically [9] as they worked at pH > pKa. 

Interestingly, cryogels prepared at pH below the pKa values demonstrated a significant shrinkage (syneresis) that indicates a high degree alginate association. A repeated freeze–thaw treatment further promoted polymer association at pH 2.5 but not at 2.0 [10]. A plausible explanation for this fact is that repeated freezing-thawing can reduce repulsive electrostatic interaction (causing gel shrinkage), whereas, at pH 2.0, the extent of aggregation is already high enough and cannot be increased further [10].

Presented results provide the first evidence for cryogelation of sodium alginate. In the context of aerogel preparation and bearing in mind a certain degree of unavoidable shrinkage upon solvent exchange and supercritical drying (see Section 6), a balance between the repulsive and attractive interactions along alginate chains should be found to obtain robust hydrogels. This can be achieved by varying the pH level and freezing-thawing regimes. Therefore, the pH level is analogous to crosslinking degree in the cryogelation process of alginate and other anionic polysaccharides.

Various binary alginate-containing blends with gelatin, PVA and other polymers also demonstrate cryogelation [11,12]. For such blends, the freeze–thawing gelation can be realized in a reversed way, resulting in so-called dual crosslinked hydrogels: alginate/PVA solution is first extruded into CaCl_2_ bath followed by several freeze–thawing cycles [13]. A ternary blend gelatin/hyaluronic acid/alginate with the cryogelation ability is also reported [14]. In all these cases, the second component is able to demonstrate cryogelation behavior on its own. Thus, the question of whether alginate plays any role in the ability to form gels upon freeze–thawing remains open.

### 2.2. Ionotropic Cryogelation

An elegant method to realize cryogelation of alginate has been described by Lozinsky [6]. This method can be seen as an extension of the classical internal setting approach where an insoluble salt is dispersed in sodium alginate and then solubilized by pH lowering. The key asset is the use of calcium salts whose aqueous solubility increases with decreasing temperature, for example calcium butyrate, pentanoate, succinate or glycerophosphate. Lozinsky reported that an aqueous solution of sodium alginate and calcium glycerophosphate (0.5 wt % each) could be crosslinked at –10 °C within 1.5 h. When cryogelation was carried out for 24 h, the tensile strength of the resulting product could be enhanced by a factor of ~3 [6]. This result suggests that the process is mass transport controlled (dissolution of the salt and diffusion of Ca^2+^ in unfrozen liquid microphase). The overall gelation kinetics is therefore influenced by the size of the salt crystals and most likely by the process temperature.

Shan et al. have applied so-called cryo-crosslinking to alginate gelation [15]. The method can be seen as a variant of the diffusion method and consists of the extruding of sodium alginate solution (2 wt %) into a bath with aqueous CaCl_2_ (25 wt %) at a temperature in the range from 0 to −15 °C. After the crosslinking, the frozen beads are filtered and thawed at room temperature.

According to the binary phase diagram for CaCl_2_—water system, the melting temperature of the 25 wt % CaCl_2_ solution is around −30 °C and thus the process takes place above the solidus line (assuming no dilution of the gelation bath). It can be assumed that, during the extrusion into cold CaCl_2_ solution, the mass transfer between droplets and the solution is limited due to fast gelation. Thus, water in the droplets should rapidly freeze. This phenomenon presumably gives rise to the macroporosity observed in the final gel. The duration of the crosslinking was not studied by Shan et al. (fixed time of 30 min in the gelation bath was used) but seems to be enough to obtain self-supporting hydrogels.

While in the work by Shan et al. [15] both freezing and gelation take place in the gelation bath, Lozinsky has suggested to spatially separate them [6]. In this method, aqueous solution of sodium alginate was frozen at a temperature below 0 °C with subsequent water removal by freeze drying or freeze–extraction. The crosslinking of water-free alginate took place in ethanolic solution of calcium acetate, resulting in a robust macroporous material (cryostructurate). An intermediate case between simultaneous and spatially separated freezing and gelation has been presented by Ho et al. [16]. In their method, aqueous sodium alginate (2 wt %) was frozen at −20 °C and then immersed into aqueous ethanol solution of CaCl_2_ at −20 °C to induce gelation of alginate.

For the preparation of macroporous alginate aerogels, the methods by Lozinsly [6] and Ho et al. [16] would have an additional important advantage: the final step takes place in ethanol or ethanol/water mixture, allowing for the elimination (at least partial) of the solvent exchange step. Moreover, water can be extracted not only by conventional solvent exchange at ambient conditions but also in the so-called freeze–extraction process, where ice is washed out by an organic solvent below 0 °C. The freeze–extraction of alginate gels (see [17]) may be of interest for low crosslinking degrees when resulting hydrogel would have low mechanical stability upon thawing. In the freeze–extraction process, the alginate network is surrounded with a non-solvent that is known to strengthen the polymer network [18]. Although attempts to apply supercritical drying to macroporous alginate structures have not yet been reported, a one-pot approach towards macroporous chitosan (cryogelation, freeze–extraction with acetone and supercritical drying) has been successfully realized [19].

## 3. Non-Solvent Induced Phase Separation

Non-solvent induced phase separation (NIPS) process (also known as immersion precipitation) dates back to the 1960s, to the work by Loeb and Sourirajan [20]. In this process, a polymer solution separates into polymer-rich and polymer-lean phases as the solubility of polymers decrease due to addition of a non-solvent. As a result, a lyogel originates that can be “hardened” by further removal of the original solvent [17]. The NIPS process is widely applied in the polymer science and technology to a diverse set of synthetic and semi-synthetic polymers. The latest achievements, the mechanisms and the key process parameters of the NIPS process have recently been summarized in a comprehensive review [21].

In the last thirty years, the non-solvents properties of organic solvents have been utilized for “hardening” of alginate and pectin hydrogel microparticles prepared by emulsion gelation [22,23]. In this context, it is important to mention the work by Oakenfull and Scott [24], who demonstrated that the gel network of high methoxyl pectin prepared in the presence of ethanol and tert-butanol is stabilized by a combination of hydrogen bonds and hydrophobic interactions. The latter are strengthened by the alcohols contributing about half to the free energy of formation of junction zones. 

**Figure 2:**
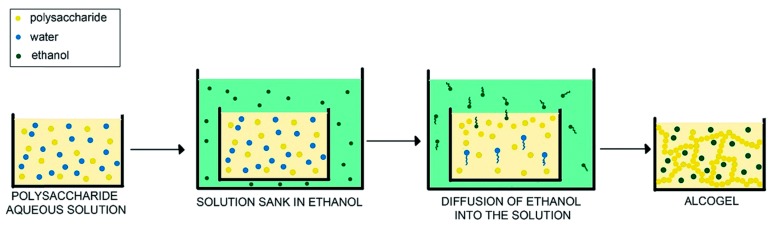
Schematic representation of the non-solvent induced phase separation process. Note that the actual volume of the alcogel is smaller due to shrinkage. Reproduced from [25] with permission of The Royal Society of Chemistry (RSC). The original RSC article is available online: http://dx.doi.org/10.1039/ 10.1039/C5RA14140K.

The NIPS process has been applied to alginate and other polysaccharides in a series of publications by Tkalec et al. [18,25,26]. Sodium alginate solution (4 wt %) without any crosslinker was exposed to ethanol for 1 or 24 h (Figure 2). Three key factors were shown to have an influence on the gel formation: nature of the non-solvent, solution viscosity (molecular weight) and duration of the process. The first factor has the most significant effect, making it clear that the polymer/solvent interactions play a very important role in the NIPS process. Alginate alcogels obtained in methanol and ethanol demonstrated much less shrinkage when compared to propanol and butanol. Remarkably, there is a close analogy between these findings and well known results in the membrane science: “frequently, the higher the mutual affinity between the solvent and non-solvent is, the more likely instantaneous demixing will occur and more porous membrane will be obtained” [21]. 

Similar observations of the solvent-specific shrinkage have been made by our group for Ca^2+^-crosslinked alginate hydrogels [27] and enzymatically (covalently) crosslinked guar galactomannan [28]. The degree of shrinkage of Ca^2+^-crosslinked alginate hydrogels in a given non-solvent was observed to be related to its hydrogen bonding component ($\delta_{h}$) of the Hansen solubility parameter: solvents with a large ability to form hydrogen bonds demonstrated less shrinkage. Thus, we believe that the phase separation in aqueous/non-solvent mixtures shares striking similarities to the well-documented shrinkage of alginate gels in organic solvents [29,30].

**Figure 3:**
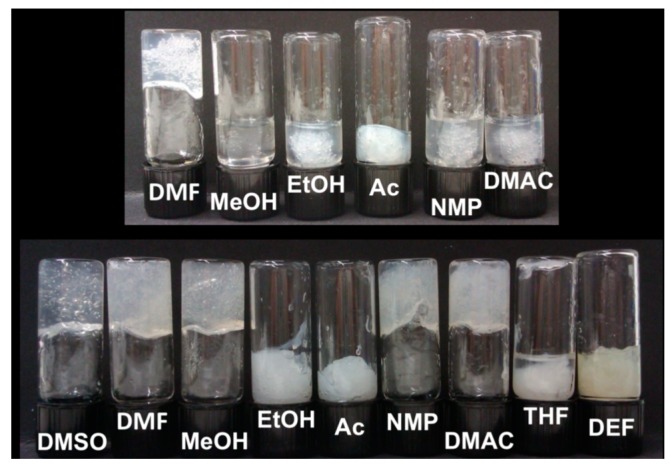
Effect of addition of 1 mL of solvents to 1 mL of 2% *w*/*v* (upper panel) or 4% *w*/*v* (lower panel) sodium alginate aqueous solution. Solvents used: dimethyl sulfoxide (DMSO), dimethylformamide (DMF), dimethylacetamide (DMAC), *N*-methyl-2-pyrrolidone (NMP), diethylformamide (DEF), tetrahydrofuran (THF), acetone (Ac), methanol (MeOH) and ethanol (EtOH). Reprinted with permission from Pérez-Madrigal et al. [31]. Copyright (2017), American Chemical Society.

Pérez-Madrigal et al. [31] have presented a systematic study of the solvent induced gelation of sodium alginate (2–8 *w*/*v* %) by adding dimethyl sulfoxide (DMSO) and other organic solvents. Supporting results from Tkalec et al. [18,25], the authors also found that aqueous sodium alginates undergo gelation upon mixing with DMSO, dimethylformamide, *N*-methyl-2-pyrrolidone, dimethylacetamide, methanol and ethanol. Gelation was shown to depend on alginate concentration: a wider range of the solvents could gel alginate solution of 4 *w*/*v* % compared with 2 *w*/*v* % (Figure 3).

Extensive molecular dynamic simulations made evident the coexistence of DMSO and water between two adjacent alginate chains: water molecules solvate the carboxylate groups, while DMSO stabilizes the ionic complexes formed between Na^+^ ions and the carboxylate groups. The hydrogen bond network formed contributes to the stabilization of the ionic complexes between charged groups [31].

Although the mechanism of the NIPS of biopolymers requires detailed studies, it is very likely that hydrophilic alginate chains contract upon adding a non-solvent. This effect has been demonstrated for dextran dissolved in good solvents [32]. The addition of a non-solvent may also lead to a lateral association of the alginate chains. The lateral association is known in Ca^2+^-crosslinked hydrogels [33] and is expected to contribute to minimizing the number of hydrophilic groups exposed to non-solvent (Figure 1b).

These two effects should result in a “packing” of several alginate chains when the thermodynamic quality of the solvent deteriorates. Results by Robitzer et al. from small-angel X-ray scattering support this, indicating that the gyration radius of Ca^2+^-crosslinked alginate hydrogel (M/G ratio 1.82) is close to 0.36 nm and decreases to 0.26 nm when water is completely replaced by ethanol [29]. The authors conclude that dehydration of the Ca^2+^-alginate hydrogel by alcohol implies a closer packing of the polymer fibrils. It is reasonable to assume that aqueous native sodium alginate should experience even a more pronounced packing. Indeed, from the SEM pictures by Tkalec et al. [18], fibrils of an aerogel precipitated in methanol have a diameter of ~55 nm while the diameter of the egg-box dimers is approx. 0.25 nm.

It is immediate from this reasoning that the degree of swelling of such packed fibrils should be lower, at least at a short time scale. This consequence has recently been demonstrated for Ca^2+^-crosslinked alginate films [34]. In this study, sodium alginate (M/G = 6:4) was crosslinked with CaCl_2_ in water/ethanol solutions (0–40 *v*/*v* %) at a constant Ca^2+^ concentration. A decreased swelling degree was measured for the films prepared at larger ethanol concentrations. Moreover, films prepared in 40 *v*/*v* % ethanol had a decreased calcium content. We interpret the results as that a partial folding of the alginate chains, which is more pronounced at high ethanol concentrations, made a fraction of carboxyl groups inaccessible, resulting in the lower crosslinking degree. Such a combined “NIPS/ionotropic gelation” may be used for cations with low affinity to alginate as demonstrated by Vicini et al. Electrospraying sodium alginate (2–3 wt %, *M*_w_ 80–120 kDa, M/G ratio 1.56) into magnesium sulfate solution in water/ethanol mixture (60:40) yielded magnesium crosslinked hydrogels [35], which are known to be unstable in water [36].

The fact that water in gels is substituted by an organic solvent directly in the NIPS process opens up attractive opportunities for aerogels processing by supercritical drying. In the above cited publications by Tkalec et al. [18,25,26], alcogels from the NIPS process were converted into aerogels by conventional drying with supercritical carbon dioxide. The surface areas and the densities were found to be by a factor of two higher than for aerogels from solvent exchanged and supercritically dried Ca^2+^-crosslinked hydrogels. This can be naturally attributed to the discussed above contraction and lateral association of the alginate chains in pure ethanol. A similar approach towards pectin aerogels has also been exemplified [26,37].

In an attempt to reduce significant shrinkage of the resulting biopolymer aerogels, one could look for a compromise and induce phase separation in water/non-solvent mixtures. An additional solvent exchange has to be performed if subsequent supercritical drying is desired [37]. Another interesting alternative is to freeze the gel with non-solvent/water mixture and perform freeze drying. Borisova et al. [38] employed the system tert-butanol/water to obtain highly mesoporous materials from alginic acid, pectin and starch. Curiously, highest pore volumes were observed when the composition was around the eutectic points of the tert-butanol/water system (23 and 90 wt %). Thereby, the NIPS process followed by the formation of fine microstructured eutectic phases upon freezing and subsequent sublimation offers an attractive route to mesoporous alginate and other polysaccharide-derived materials. 

## 4. Carbon Dioxide Induced Gelation

**Figure 4:**
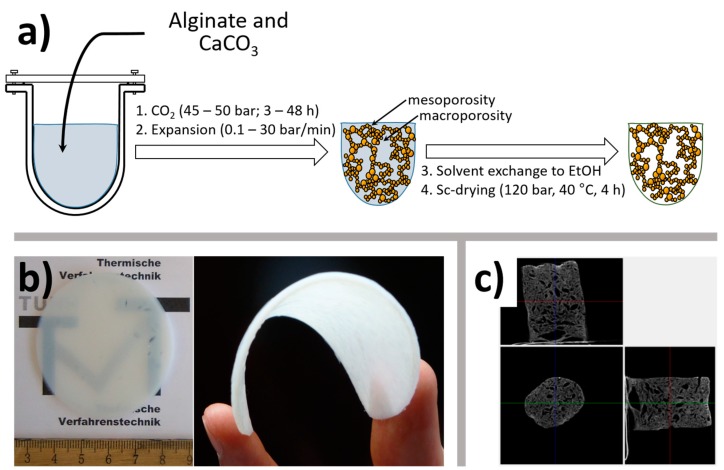
(**a**) general scheme of the carbon dioxide induced gelation; (**b**) as synthesized translucent monolithic alginate aerogel from 0.5 wt % sodium alginate solution (left panel), alginate aerogel after compression under a pressure of 10 kN/m^2^ becomes flexible; (**c**) microtomography image of alginate–lignin aerogels produced by CO_2_ induced gelation depressurized at 30 bar/min to introduce macropores. Part (**a**) is reprinted from Martins et al. [42], Copyright (2015), with permission from Elsevier; part (**b**) is reproduced from Gurikov et al. [39] with permission from the Royal Society of Chemistry; part (**c**) is reprinted from Quraishi et al. [43], Copyright (2015), with permission from Elsevier.

There are many ways to trigger a release of cations that is present in insoluble or chelated form. Our group has recently developed the so-called carbon dioxide induced gelation [39,40]. In this method, a suspension of metal carbonate or hydroxycarbonate (Ca, Sr, Ba, Zn, Cu, Ni or Co) in aqueous sodium alginate is subjected to a pressurized carbon dioxide (30–50 bar) at room temperature (Figure 4a). Although first attempts to gel alginate/CaCO_3_ suspension in carbon dioxide atmosphere were made by Draget et al. in 1990, the process was performed at 1 bar and thus no gelation has been observed within 48 h for a 8 mm slice due to extremely slow mass transfer [41].

This method is applicable to a wide range of alginate blends with other bio- and water soluble synthetic polymers and can also be used to entrap insoluble microparticles (“alginate as a glue”). An interesting feature of the carbon dioxide induced gelation that a significant amount of CO_2_ is dissolved in aqueous phase of the gel at the end of the process. Depending on the depressurization rate and gel thickness, hydrogels with macrosized voids can be prepared [42,43]. Such foamed hydrogels are free of templating agents and may be an interesting scaffold for biomedical applications. Conceptually, a similar approach has been described by Barbetta et al. [44], where in situ formed CO_2_ bubbles (reaction between NaHCO_3_ and citric acid) were entrapped in the gel upon crosslinking with CaCl_2_. Another positive aspect of the CO_2_ induced gelation is that even large monoliths can be easily prepared [45].

One underlying motivation for the development of the carbon dioxide induced gelation is to reduce the number of steps in aerogel production as far as possible by combining gelation, solvent exchange and sc-drying into an integrated approach using CO_2_. Such an integration has been demonstrated [39,46]: the alginate/CaCO_3_ suspension was first gelled in CO_2_, and then the pressure and temperature was raised to 120 bar and 45 °C followed by the solvent exchange at these conditions by introducing ethanol/water mixtures with a high pressure pump directly into a high pressure autoclave. Once the solvent exchange was finished, the gels were dried with pure supercritical CO_2_.

We found that the alginate aerogels obtained via carbon dioxide induced gelation route (Figure 4b) have exceptionally high specific surface areas and volume of mesopores. Although further studies are needed, we surmise that even slow depressurization leads to a certain expansion of the hydrogel backbone. Typically, around 50% of the overall porosity can be attributed to the volume of mesopores (2–50 nm) [39].

Mechanistic explanation of the carbon dioxide relies on the increase of CO_2_ solubility in water with rising pressure along with lowering of pH down to 3. The drop in pH causes in turn an increase in solubility of calcium carbonate and thereby triggers a release of calcium ions. In typical conditions (25 °C and 40–50 bar), CaCO_3_ solubility is ca. 2.8 g/L compared to 0.006–0.01 g/L at ambient conditions [43]. Thus, a considerable amount of Ca^2+^ is available for the reaction with alginate. It is important to mention that experiments in a tilting viewing high pressure cell showed neither gelation nor even a noticeable increase in viscosity of plain sodium alginate solution.

The fact that textural properties of the alginate aerogels derived from hydrogels via CO_2_ induced were found to be superior to those from conventional processes, the role of pressurized carbon in the gelation process requires further studies. We briefly mention here that other biopolymers can also be gelled in pressurized carbon dioxide, e.g., silk protein [47]. The results of Floren et al. [47] indicate that silk protein hydrogels prepared under CO_2_ pressure followed by slow depressurization display distinctly more homogeneous pore structure compared to hydrogels acidified by citric acid at ambient conditions. This result clearly shows that carbon dioxide induced gelation, not followed by fast depressurization, leads to more compact hydrogels compared to ambient conditions. This conclusion is in agreement with observations made by Annabi et al. for elastin-based hydrogels [48].

## 5. Other Gelation Methods

An interesting finding has been reported by Pérez-Madrigal et al. [31]. In addition to the results on the non-solvent induced phase separation (Section 3), the authors demonstrated that carboxylic acids such as oxalic, maleic, tartaric, glutaric and citric acids (pH 1.0–2.0) can also induce gelation of sodium alginate. Gelation with oxalic, citric and maleic acids was found to be fast so that gel beads could be prepared by extruding alginate solution (4 *w*/*v* %) into the corresponding acidic solution (0.5 M). No gelation was detected in blank experiments with acetic acid. Interestingly, numerous washings of carboxylic acid-crosslinked gels with water to a neutral pH did not lead to gel disintegration. These results clearly indicate a strong interaction between the acid and the alginate chains. Such interactions have also been evidenced by density-functional theory DFT calculations and Langevin molecular dynamics [31].

**Figure 5:**
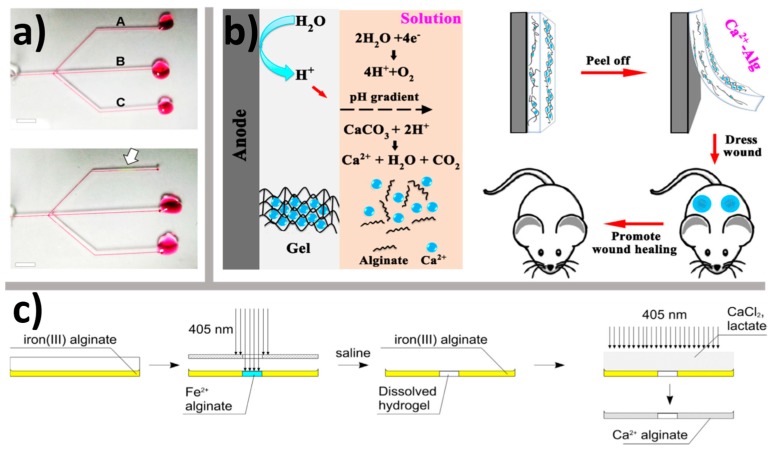
(**a**) localized flow-blocking by UV induced gelation of alginate; upper panel: the fluid flows freely through all three channels (A, B and C); lower panel: gel is formed (indicated by arrow) in the channel A upon UV exposure; (**b**) electrodeposition of calcium-crosslinked alginate film triggered by the release of Ca^2+^ ions from insoluble CaCO_3_ particles; (**c**) preparation of patterned calcium crosslinked alginate hydrogel by photochemical patterning of iron (III) crosslinked alginate hydrogel, dissolution of the exposed areas and photochemical reductive cation exchange. See text for details. Part (**a**) is adapted with permission from Oh et al. [49], Copyright (2016), American Chemical Society; part (**b**) is reproduced by permission from Liu et al. [50], Copyright (2017), Springer Nature; part (**c**) is adapted from Bruchet and Melman [51], Copyright (2015), with permission from Elsevier.

Release of crosslinking cations in the internal setting method is usually triggered chemically, most often by adding slowly hydrolyzing GDL. An interesting alternative is to release cations is to use photoacid generator (diphenyliodonium nitrate) in a combination with methyl-β-cyclodextrin (mβ-CDs) [49]. Strontium chelate of ethylene glycol tetraacetic acid added to sodium alginate (2.5 wt %, *M*_w_ 500 kDa) reacts with photoacid generator upon exposure to UV light. The reaction liberates Sr^2+^ cations that crosslink alginate, while byproducts of the photolysis are sequestered by mβ-CDs, Figure 5a. 

Another approach is to generate protons by electrolysis of water in the aqueous solution of sodium alginate. Protons generated on anodes diffuse into the bulk phase and liberate Ca^2+^ from dispersed CaCO_3_ particles, resulting in film deposition [50], Figure 5b).

Preparation of Ca^2+^-crosslinked hydrogels can be realized using Fe^3+^-crosslinked alginate hydrogels as a starting material through UV induced reduction of Fe^3+^ into Fe^2+^ [51]. Because Fe^3+^ has a higher affinity to alginate than Ca^2+^, direct exposure to CaCl_2_ solution does not result in appreciable ion exchange. During photochemical reduction by a reaction with lactic or 2-hydroxybutyric acid in CaCl_2_ solution cations Fe^2+^ can be substituted by Ca^2+^. The use of a sacrificial photoreductant such as lactic acid gives a better control over ion exchange process and allows for photochemical patterning [51], Figure 5c. 

Metal-crosslinked alginate beads can also be used for crosslinking fresh portions of alginate. Vicini et al. have crosslinked sodium alginate (2–3 wt %, *M*_w_ 80–120 kDa, M/G ratio 1.56) by electrospinning into concentrated solutions of barium, calcium and magnesium salts. The beads were then solvent exchanged with ethanol and dried.

## 6. Drying of Alginate Gels

A wide range of gelation techniques was discussed in the previous sections. When a solid material is desired, gel has to be dried. There are three ways to remove solvent from a gel: evaporative drying, freeze drying and supercritical drying. Despite a range of practical implementations, from a fundamental perspective, the evaporative drying deals with the mass transfer from the liquid phase (solvent in gel) to the gaseous phase. In the freeze drying process, the solvent has to be first frozen and then sublimes (at ambient or reduced pressure), leaving behind a solid material (often called cryogels, not to be confused with gels obtained by cryogelation, see Section 2). Supercritical drying makes use of the fact that there is a region on the (p,T)-diagram where the solvent is in the supercritical state. The resulting material is called aerogel.

Evaporative drying may often be an obstacle towards porous materials, as considerable capillary forces lead to the structure collapse and apparent shrinkage. This effect is very pronounced for alginate and other polysaccharides due to their hydrophilic nature and can be eliminated by grafting of hydrophobic groups. Although hydrophobization followed by evaporative drying has been shown as a viable approach for drying of cellulose gels [52], it has not yet applied to alginate gels, to the best of our knowledge.

Two remaining drying methods do not yield materials with identical morphology. Freeze drying of hydrogels is a routinely used procedure to obtain materials with distinct slit and interconnected macropores and very little fraction of mesopores. Such morphology stems from the fact that the porous structure of hydrogels undergoes considerable changes due to growth of ice crystals. It is known that freezing regimes have a great influence on the pore morphology [53,54]. To obtain mesoporous alginate with appreciable surface area, a special technique is required such as aforementioned sublimation of fine crystals of tert-butanol/water eutectic mixture [38]. Although in the vast majority of cases water is the most natural solvent to be removed, the use of non-aqueous solvents and binary mixtures may be worth the effort [55,56].

Supercritical drying of alginate and other polysaccharide gels has been studied in more detail (see [30,57] and references therein). Due to very low solubility of water in supercritical carbon dioxide, alginate hydrogels cannot be dried immediately: water has to be substituted by an organic solvent. As we discuss in Section 3, not every solvent is equally compatible with hydrogels: solvent exchange with nonpolar aprotic solvents results in a severe shrinkage. Preference should be given to methanol, ethanol and DMSO [27]. Stepwise solvent exchange with a progressively increasing concentration of the solvent is a well known technique to minimize shrinkage [29].

Alginate aerogels are unique mesoporous materials in terms of specific surface area and pore volume, 400–600 m^2^/g and 3–10 cm^3^/g, respectively. Thus, supercritical drying is a milder technique to retain delicate gel structures [29]. It is worth noting that macroporosity introduced into intrinsically mesoporous gels is also retained upon supercritical drying (see Figure 4c). Alginate aerogels also demonstrate low thermal conductivity (superinsulation), high water uptake and can be loaded with various active ingredients from sc-CO_2_ after the drying [43,45,58]. 

As discussed above, alginate gels obtained from the non-solvent induced phase separation process can be supercritically dried without the solvent exchange. Gelation of native alginate in non-aqueous solvents, which would allow for immediate supercritical drying, is unknown and can hardly be imagined.

Overall, two well established drying approaches, freeze and supercritical drying, can follow the gelation when solid porous alginate matrices are desired. Attempts to relate the drying method to pore morphology still remain to be made for alginate. Recent works on cellulose [59,60] shed more light on how such studies can be designed.

## 7. Perspectives 

In the present review, we described recently reported non-conventional approaches for gelation of native alginate. These approaches are far from being understood at both molecular and engineering levels. Nevertheless, we believe that they offer attractive and, with further developments, powerful additions and alternatives to the existing and better studied methods.

Well known for synthetic polymers, there is only limited knowledge on cryogelation of anionic polysaccharides. It is evident that partial protonation of the polymer chains favors stabilization of junction zones upon thawing. An optimal pH value for cryogelation of alginate, the influence of the G/M ratio and the number of freezing-thawing cycles need to be studied. At a molecular level, the nature of the junction zones and the role of hydrogen bonding in alginate cryogels needs to be studied. To obtain solid materials, the freezing–thawing approach can naturally be combined with freeze drying. Research on interrelations between the structures of parent and resulting gels depending on regimes of the freeze drying represents significant opportunities.

Non-solvent induced phase separation offers an attractive alternative towards lyogels free of crosslinking cations. Such lyogels can be further converted into aerogels. In this way, the gelation and the solvent exchange can be collapsed into a single step. The morphology at meso- and macroscale are merely determined by nature of the non-solvent. Deeper understanding of the phase separation and the role of the polymer/non-solvent interactions is needed. Measurements of the Hansen solubility parameters for alginates and the application of the Flory–Huggins model may be a first step towards this goal. This knowledge in combination with an efficient supercritical drying protocol may pave the way for potential industrial applications. 

Rational pore engineering in wet and dry states still remains a challenge in material science. The methods discussed in the review broaden the range of tools and tactics for the pore engineering. The authors hope that these methods will inspire and motivate researchers from different areas to pursue activities in this field.
